# Experimental determination of the velocity distribution in USP Apparatus 1 (basket apparatus) using Particle Image Velocimetry (PIV)

**DOI:** 10.1016/j.ijpx.2021.100078

**Published:** 2021-05-04

**Authors:** Chadakarn Sirasitthichoke, Satish Perivilli, Mark R. Liddell, Piero M. Armenante

**Affiliations:** a New Jersey Institute of Technology, Otto H. York Department of Chemical and Materials Engineering, Newark*,* NJ 07102-1982*,* USA; b United States Pharmacopeial Convention (USP), Dosage Form Performance Laboratory (DFPL), Rockville, MD 20852-1790, USA

**Keywords:** Drug dissolution, USP apparatus 1 (basket), Hydrodynamics, Particle image velocimetry, Dissolution testing

## Abstract

The USP Apparatus 1 (basket apparatus) is commonly used to evaluate the dissolution performance of oral solid dosage forms. The hydrodynamics generated by the basket contributes, in general, to the dissolution rate and hence the dissolution results. Here, the hydrodynamics of Apparatus 1 was quantified in a vessel filled with 900-mL de-ionized water at room temperature by determining, via Particle Image Velocimetry (PIV), the velocity profiles on a vertical central plane and on 11 horizontal planes at different elevations at three different basket agitation speeds. The flow field was dominated by the tangential velocity component and was approximately symmetrical in all cases. Despite all precautions taken, small flow asymmetries were observed in the axial and radial directions. This appears to be an unavoidable characteristic of the flow in Apparatus 1. The magnitudes of the axial and radial velocity components varied with location but were always low. A small jet was seen emanating radially near the top edge of the basket. Velocities typically scaled well with increasing agitation speed in most regions of the vessel except for a region directly below the basket. The results of this work provide a major insight into the flow field inside the USP Apparatus 1.

## Highlights

• Velocity profiles in the USP Apparatus 1 (basket) were obtained using PIV.
• Velocity profiles are very sensitive to small deviations from perfect symmetry.
• Flow throughout the vessel is dominated by the tangential velocity component.
• The axial and radial velocity components are relatively weak throughout the vessel.
• A small radial jet near the basket top contributes to forming recirculation loops.

## Graphical abstract

**Figure:**
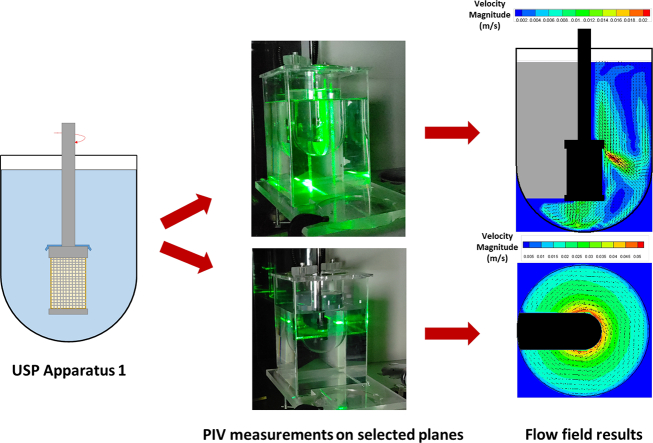

## Notation

*D*: basket diameter, m or mm
*H*: liquid height, m or mm
*N*: basket agitation (rotational) speed, revolutions per minute (rpm) or
  revolutions per second (rps)
Δ*l*: thickness of laser sheet, m or mm
*r*: radial coordinate of PIV measurement point, m or mm
*R*: radius of the dissolution vessel, m or mm
*T*: internal diameter of vessel, m or mm
*U*_*a*_: velocity in the axial direction, m/s
*U*_*r*_: velocity in the radial direction, m/s
*U*_*t*_: velocity in the tangential direction, m/s
*U*_*tip*_: basket tip velocity, m/s
*V*: fill volume, mL
*Y*: vertical location of horizontal isosurface, m or mm
Δ*t*: time difference between two laser pulses, s or ms

**Greek symbols**
*μ*: liquid viscosity, kg/(m∙s)
*ρ*: liquid density, kg/m^3^

**Non-dimensional groups**
*Re*: Reynolds number *=* *ρND*^*2*^*/μ*
*St*: Stokes number = Δ*t∙U*_*t*_*/*Δ*l*

## 1 Introduction

In the pharmaceutical industry, drug dissolution testing is routinely carried out in-vitro in order to provide critical information about drug dissolution in-vivo, particularly in cases where the dissolution, with the consequent release of the drug from the formulation matrix, is the rate limiting step in drug absorption. In addition, drug dissolution testing is used to assess drug products in stability studies to support drug product approvals, to assess batch-to-batch reproducibility during production as a quality control activity, and/or to evaluate the impact of post-approval manufacturing changes, as required by the United States Food and Drug Administration (FDA) (U.S. Department of Health and Human Services, 1995).

Dissolution testing is typically conducted using standard apparatuses and methods detailed in the United States Pharmacopoeia (USP-NF) (United States Pharmacopeial Convention, 2018). USP develops and disseminates compendial quality standards for drug products and other pharmaceutical products, including the dissolution testing methods accepted by the FDA (U.S. Department of Health and Human Services, 1997). USP dissolution testing apparatuses are used as analytical testing equipment to evaluate the performance of a drug product and to indicate whether the drug product performs within the acceptance standard criteria (United States Pharmacopeial Convention, 2018).

Several reports in the literature have indicated that the distribution of fluid velocity profiles obtained in dissolution testing apparatuses (i.e., those included as dissolution apparatuses in USP General Chapter <711>) varies from one apparatus to another and also varies spatially within an apparatus (for example, with Apparatus 2) (Bai and Armenante, 2008; Bai et al., 2007; D'Arcy et al., 2006; McCarthy et al., 2004; McCarthy et al., 2003). This is to be expected, since the hydrodynamics of different dissolution testing apparatuses is dependent on geometries and operation methods of the apparatuses (Ameur and Bouzit, 2013). Significant differences in the dissolution profiles of controlled-release tablets were found depending on the type of apparatus and operating conditions (Lu and Fassihi, 2017). Additionally, the tablet is placed at different locations in each apparatus (e.g., inside the basket in USP Apparatus 1 vs. below the paddle in USP Apparatus 2). Dissolution might also be influenced by where the dosage form or its fragments are located relative to fluid velocity distribution within the apparatus. The influence of tablet location on the hydrodynamics and associated mass transfer and dissolution rates was reported for USP Apparatus 2 (Bai and Armenante, 2009). Within these considerations, results from these dissolution apparatuses can be highly variable and may contribute to difficulty meeting product specifications.

Having a sound understanding of the hydrodynamics in the in-vitro dissolution testing apparatus could help in better understanding the in-vitro dissolution behavior of the dosage form. In fact, the drug dissolution profile obtained during a typical drug dissolution test is the result of a complex combination of a number of factors, including several factors not directly related to the system hydrodynamics, such as drug formulation and drug release mechanism, media degassing and preparation, sample filtering, and those that can influence the hydrodynamics such as external vibrations, set up of the instrument, placement of the dosage form, and others. The critical role of hydrodynamics in the dissolution process was clearly evidenced in previous studies conducted with USP Apparatus 2 (Bai and Armenante, 2009; Wang et al., 2018). According to the literature, small changes in the geometry of this apparatus can possibly contribute to increasing the level of variability in dissolution testing processes and test results (Cox and Furman, 1982; Cox et al., 1983). The prevailing hydrodynamics in this dissolution apparatus appears to be sensitive to any changes that may be inadvertently introduced by the operator or may be the result of the protracted use of the apparatus and associated wear-and-tear (Wang and Armenante, 2012). These considerations likely apply to all apparatuses, including USP Apparatus 1, although the literature on this apparatus is less abundant than for USP Apparatus 2.

The USP Apparatus 1 (basket) is one of the most commonly used compendial dissolution testing apparatuses in the pharmaceutical industry. The USP Apparatus 1 consists of a wire basket, typically 40 mesh, rotating at a constant speed (between 50 and 100 rpm), in an appropriate medium (500-mL or 900-mL fill volume) contained in a glass vessel. A solid dosage unit is placed in the basket and the amount of drug substance dissolved over time is determined. The exact physical dimensions of the different components of the whole assembly and the basket motion can strongly affect the drug dissolution rate.

Despite its widespread use in the pharmaceutical industry, very limited information is available in literature on the hydrodynamics of USP Apparatus 1, both in terms of experimental data and computational results. Diebold and Dressman (2001) used an ultrasound-pulse-echo measurement to determine the velocity magnitude but only at specific locations away from the basket. D'Arcy et al. (2006) predicted the hydrodynamics in Apparatus 1 using a Computational Fluid Dynamics (CFD) approach. They found that the basket creates a weak recirculation flow in the lower portion of the vessel resulting in an upward axial flow which enters the basket from the bottom and then leaves it, radially, at the lower bottom side of the basket. The highest velocities that they predicted were found along the basket sides with intermediate velocities extending toward the vessel wall, while low velocities were predicted everywhere else in the vessel. More recently, Martinez et al. (2020) conducted CFD simulations at various agitation speeds (50–200 rpm) in a dissolution vessel with a 250-mL liquid fill volume using a 10-mesh basket size with a 6.8-mm clearance between the bottom of the basket and vessel. In addition, they experimentally observed the dispersion of a dye contained within the basket and visually found that the dye was released outward from the sides of the basket into the fluid bulk and then recirculated back into the basket from the basket base. They found that their visual observations were in good overall agreement with their own CFD results. However, their comparison was exclusively qualitative, and their results were obtained with a dissolution system that is not compendial and not typically used in dissolution testing.

This review shows that, to the best of our knowledge, there is a need for additional work on the hydrodynamics on USP Apparatus 1, especially as far as the experimental determination of the flow field inside the apparatus is concerned. While CFD may be more economical and less time-consuming, experimental quantification of the hydrodynamics is always essential not only to understand the flow behavior of the system but also to validate computational predictions. Various experimental methods have been used in the past to quantify the velocity flow field in stirred tanks and vessels, such as Laser-Doppler Anemometry (LDA), Planar Laser-Induced Fluorescence (PLIF) and Particle Image Velocimetry (PIV) (Bai et al., 2007; Li et al., 2004; Liu et al., 2016; Liu et al., 2017; Montante et al., 2006; Montante et al., 2001; Motamedvaziri and Armenante, 2012; Ng et al., 1998; Stamatopoulos et al., 2015; Trad et al., 2017). PIV has also been shown to be a valuable technique to determine instantaneous field of the velocity vectors and gain insight into flow features in dissolution apparatuses (Kukura et al., 2003; Perivilli et al., 2017; Wang et al., 2018; Yoshida et al., 2015). Experimental techniques such as PIV also have their own limitations because of the intrinsic difficulty to access areas of interest that may be inaccessible experimentally, such as the inside of the basket in USP Apparatus 1. However, even in this case, quantification of the flow profiles outside the basket can provide critical information about influx/outflux of fluid into/out of the basket, can characterize mixing regions outside the basket, and can provide information of transport phenomena expected for the solution outside the basket. Furthermore, experimental measurements are essential to validate computational models which can then provide information on the flow inside the basket without having the limitations of the experimental techniques.

Therefore, in this work the velocity distribution in the USP Apparatus 1 was experimentally quantified using 2-dimensional, 2-component (2D-2C) PIV. The specific objective of this work was to obtain detailed maps of the flow field in this apparatus and fully quantify the velocity profiles in the standard USP Apparatus 1 at different basket agitation speeds, as described in the USP-NF. Detailed, point-by-point measurements of all three velocity components, i.e., tangential, radial, and axial, were obtained throughout the vessel, and comparisons were made using a non-dimensional approach to better understand the effect of the agitation speed on the velocity profiles and to provide a critical insight into the flow moving through the basket.

## 2 Experimental apparatus, material and method

### 2.1 Dissolution vessel and agitation system

**Fig. 1:**
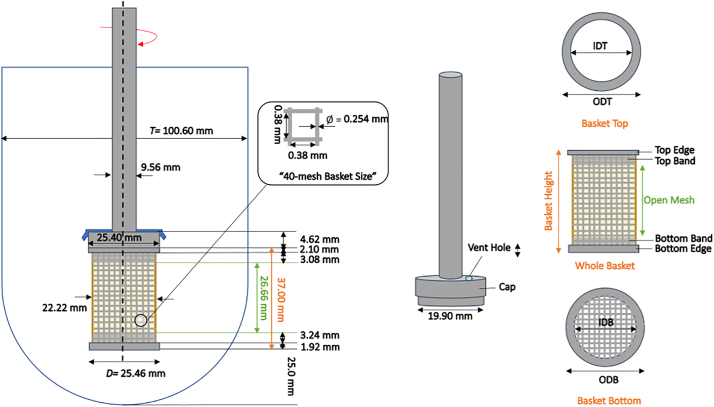
Basic geometry of USP Apparatus 1 vessel and basket (dimensions not to scale; see Table 1 for additional dimensions and acronyms).

**Fig. 2:**
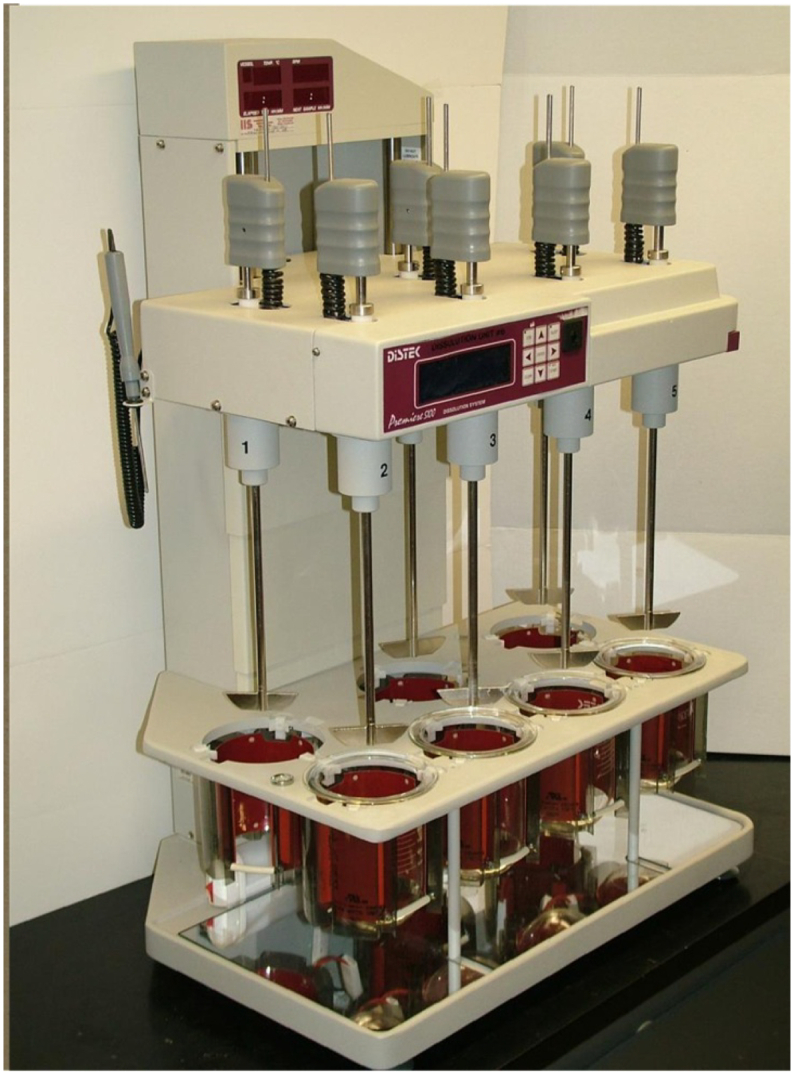
Distek™ Evolution 6100 Bathless Dissolution System used in this work.

**Fig. 3:**
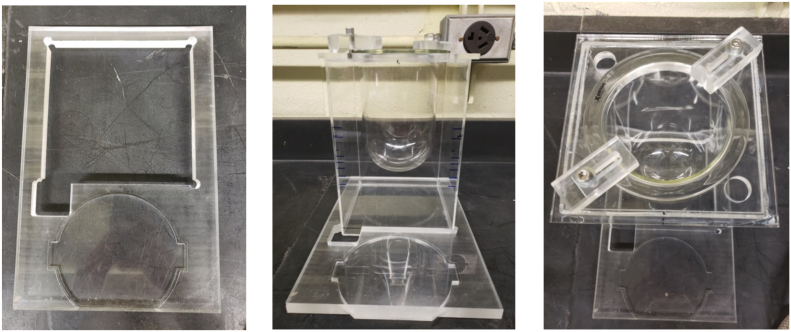
Custom-made plexiglass square tank base (left panel), and square tank/vessel assembly (center and right panel).

**Fig. 4:**
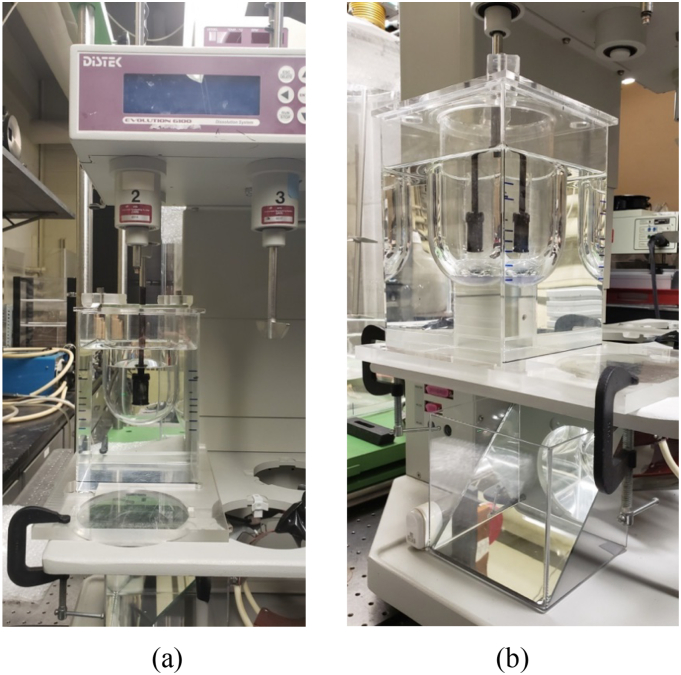
Dissolution testing unit (Distek™ Evolution 6100 Bathless Dissolution System) appropriately fitted with the dissolution vessel/square tank/base assembly: (a) front view; (b) side view.

A standard USP Apparatus 1 glass vessel (Jasco, Inc.) consisting of an unbaffled cylindrical glass vessel (internal diameter, *T* = 100.6 mm) with a hemispherical bottom and an overall capacity of 1 L was used in all experiments (Fig. 1). Agitation was provided by a Distek™ Evolution 6100 Bathless Dissolution System (Distek Inc., North Brunswick, NJ, USA), shown in Fig. 2. However, this system was not assembled as typically done in a standard dissolution test. Instead, a custom-made plexiglass holding tray assembly (Fig. 3) was used to hold the vessel in the correct position in the Distek™ system and obtain velocity measurements, as described below. The assembly consisted of a plexiglass square tank provided with a lid in which a hole having a diameter equal to the outer diameter of the dissolution vessel was precisely drilled, so that the dissolution vessel could be held, suspended from the top, inside it. The square tank fitted precisely in a custom-made plexiglass base. This whole assembly could hold the dissolution vessel inside the square plexiglass tank and, because of the fitted base, exactly mount the dissolution vessel/square tank assembly in a pre-determined fixed position *above* the heavy-duty steel plate (vessel plate) of the Distek™ system (i.e., *not* inside the round openings in the vessel plate where vessels are typically inserted during dissolution tests), as shown in Fig. 4. This arrangement ensured that the dissolution vessel would be precisely centered under one of the agitation shafts of the Distek™ apparatus (to enable basket rotation) while, enabling viewing of the dissolution vessel from the front, side, and bottom (thus allowing velocity measurements to be taken in three directions using the PIV system). A separate square plexiglass box containing a mirror placed at 45° angle was constructed (Fig. 4(b)) and placed below the custom-made dissolution vessel assembly in the Distek™ system so as to allow viewing the bottom of the dissolution vessel and taking PIV velocity measurements on horizontal cross sections inside the vessel.

**Fig. 5:**
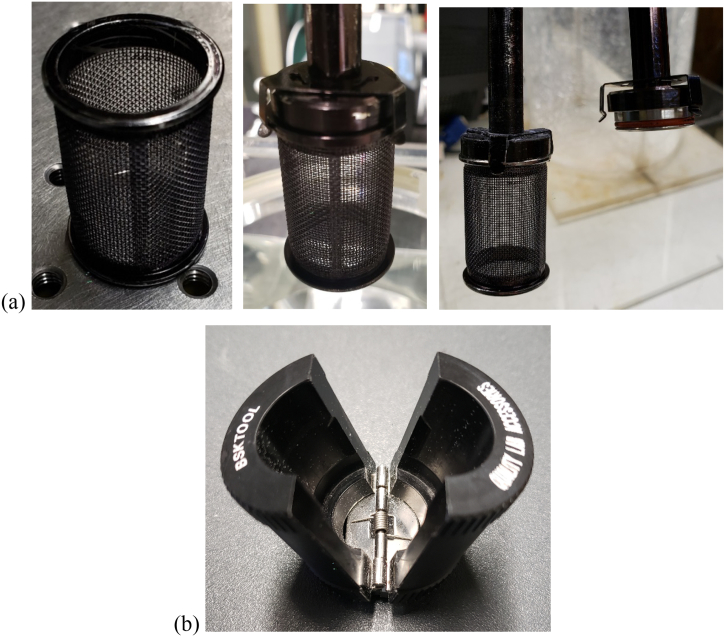
(a) Dissolution basket (left panel) and details of the three-pronged clip securing the basket to the shaft (center and right panels); (b) Basket grabber (QLA).

The agitation system used in this study consisted of a dissolution basket (40-mesh, stainless-steel basket, Quality Lab Accessories (QLA), Telford, PA, USA) and a stainless-steel shaft connected to one of the motors in Distek™ system. The dimensions of the basket were obtained with a Vernier caliper and are reported in Fig. 1 and Table 1. The same brand-new basket was used in all experiments to ensure that any imperfection in the basket or any basket wobbling effects could not be attributed to poor handling of the basket. Therefore, the “basket grabber” shown in Fig. 5b (QLA) was used to handle the basket and mount it on the shaft without touching the basket mesh.

**Table 1:** Shaft and basket dimensions (geometric definitions are provided in Fig. 1.)

Components	Dimension (mm)
Shaft diameter	9.56
Outside diameter of cylindrical wire mesh screen	22.22
Inner diameter of basket top edge (IDT)	20.20
Outer diameter of basket top edge (ODT)	25.40
Inner diameter of basket bottom edge (IDB)	20.60
Outer diameter of basket bottom edge (ODB)*	25.46
Thickness of top band	0.30
Height of top band	3.08
Thickness of bottom band	0.30
Height of bottom band	3.24
Height of open mesh	26.66
Height of top edge	2.10
Height of bottom edge	1.92
Basket height	37.00
Mesh screen openings	40 mesh
Size of square openings	0.38
Wire diameter	0.254
Height of cap	4.62
Vent hole diameter	2.06

(*) Used in the calculation of basket tip speed, *U*_*tip*_: $U_{\mathrm{tip}}\;\left(\mathrm{in}\frac{\mathrm{mm}}{s}\right)=\pi \times \frac{N\;\left(\mathrm{in}\;\mathrm{rpm}\right)}{60}\times \mathrm{ODB}\;\left(\mathrm{in}\;\mathrm{mm}\right)$

As specified in USP-NF, the basket was secured to the shaft using a three-pronged metal clip that was permanently attached to the shaft. The dissolution basket and some parts of the vertical shaft were lightly colored with a permanent black marker pen so that the laser sheet would not be reflected by these devices. The thickness of the ink coat on the dissolution basket was negligible since a black marker pen was used instead of paint, as shown in Fig. 5a.

### 2.2 Particle image velocimetry (PIV) system

**Fig. 6:**
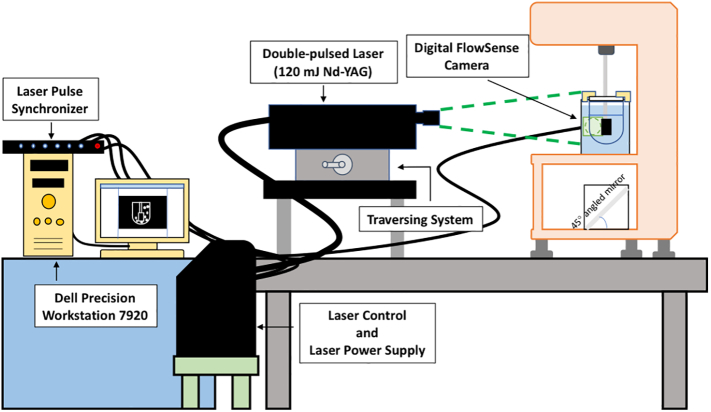
Schematic of laboratory PIV experimental set-up.

A schematic diagram of the experimental apparatus is shown in Fig. 6. The PIV system (Dantec Dynamics, Denmark) used to experimentally measure the flow field and velocity distribution on the selected plane in the present study consisted of five components: (a) double pulsed 120 mJ Nd-YAG laser (model: Solo 120 15 HZ, New Wave Research, Inc., Fremont, CA, USA), (b) Laser Power Supply (model: Solo 120 15 Hz, New Wave Research, Inc), (c) digital high-resolution FlowSense VCXU 2 M-165 (1920 × 1200 pixels) CMOS camera (Dantec Dynamics), (d) synchronization unit (time resolution <8 ns; 8 outputs, 2 inputs; Dantec Dynamics), and (e) computer (Dell Precision WorkStation 7920, Six Core Intel® Xeon® Bronze 3104 CPU @ 1.70 GHz) for data acquisition and data analysis.

The laser system consisted of two infrared laser heads combined in a single package with a second harmonic generator and two discrete power supplies. The laser light source came from a Class IV laser generating two pulsed infrared laser beams with a wavelength of 1064 nm that passed through an optical arrangement and an optical crystal to convert most of the infrared light to a visible green laser light sheet emitted at 532 nm. The light sheet thickness (Δ*l)* was always 4 mm. The time difference between two laser pulses, Δ*t*, was a critical data acquisition parameter which was selected to optimize the displacements of the particles. In addition, the size of the interrogation windows and thickness of the laser light sheet were chosen so that the displacements of particles traveling through the laser sheet could be captured during that Δ*t* with minimal loss-of-pairs data and could therefore be used to determine the velocity at that location.

### 2.3 Materials

Minute amounts (5 ± 0.5 mg) of polyamide seeding particles (PSP, Dantec Dynamics, Denmark) with a particle size of about 50 μm and a density of 1.03 g/cm^3^ were used as tracer particles because they were large enough to scatter the light from the laser sheet, making them visible to the FlowSense camera, but small enough to follow the fluid motion without affecting the flow (i.e., with Stokes Number, *St* < <1). A fixed amount (900 mL) of de-ionized (DI) water at room temperature was used as the dissolution medium in all experiments. This temperature was different from the USP specifications (37 ± 0.5 °C) because it would have been very difficult to thermostat the USP vessel-square tank assembly, maintain precise alignment of all components, and, at the same time, capture images on both the horizontal and vertical planes during the PIV experiments. While it was recognized that there are differences in fluid properties due to the temperature difference, the objective of this study was to characterize the hydrodynamics in the Apparatus 1 as defined by its geometry and operation (i.e., changes in agitation speed). A trace amount of Polyoxyethylenesorbitan monooleate (Tween 80, Sigma-Aldrich 9005-65-6) was used as a surfactant in order to wet the tracer particles.

### 2.4 Experimental procedure

In order to precisely align the different components of dissolution apparatus (vessel, shaft, etc.) and fully obtain accurate velocity results in the USP Apparatus 1, the system was firstly calibrated using specifications in the USP dissolution toolkit, FDA guidances, and ASTM specifications (referred to as “standard calibration” from here on). The geometric tolerances were measured to be within the limits specified by those organizations/agencies, as detailed in Table 2.

**Table 2:** Geometric tolerances in the alignment of the components of dissolution testing apparatuses from USP “Dissolution Toolkit Procedures for Mechanical Calibration and Performance Verification Test Apparatus 1 and Apparatus 2. Version 2.0”, FDA Guidance for industry “The Use of Mechanical Calibration of Dissolution Apparatus 1 and 2 – Current Good Manufacturing Practice (CGMP),” DPA-LOP.002, and ASTM (Standard E2503-13e1) “Standard Practice for Qualification of Basket and Paddle Dissolution Apparatus.”

	USP	FDA	ASTM
Shaft Verticality	Not specified	Vertical in 2 directions 90° apart. ≤0.5° from vertical	Within bubble in the bubble level
Centering	≤2 mm from center axis	≤1.0 mm of center line	<1.0 mm from centerline of shaft or surrogate shaft
Wobble	No significant wobble for shaft and ± 1 mm for basket wobble	≤1 mm total runout of shaft measured 2 cm above basket or blade and ≤ 1 mm total runout of basket measured at the bottom edge of the basket	

**Fig. 7:**
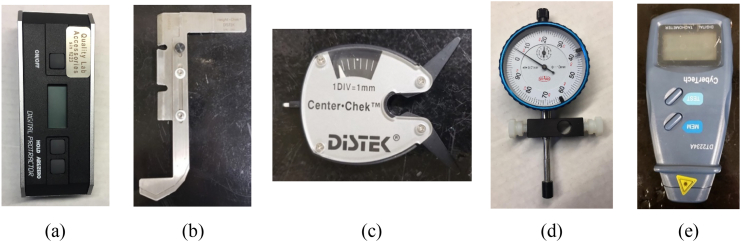
Dissolution toolkits were used to support this experimental study: (a) digital protractor; (b) HeightChek™; (c) CenterChek™; (d) wobble meter; (e) laser photo tachometer.

The dissolution toolkit consisted of four instruments, as shown in Fig. 7a–d, i.e., (a) digital protractor (QLA, Telford, PA, USA) to check the verticality of shaft, (b) HeightChek ™ (QLA, Telford, PA, USA) to set the basket clearance off the vessel bottom to 25 mm prior to a test, (c) wobble meter (QLA, Telford, PA, USA) to check the centering and wobbling of the dissolution basket, (d) CenterChek™ (DISTEK™ model 170, Distek Inc., North Brunswick, NJ, USA) to align the centerline of the shaft holding the basket within the vessel centerline. In addition, a digital photo laser tachometer was used to verify the accuracy of the rotational speed (CyberTech, Chino, CA, USA).

Initial preliminary PIV experiments showed that the system was extremely sensitive to minor geometric variations. Therefore, the system was re-adjusted following much more stringent requirements (referred to as “improved calibration” or “precise alignment” from here on) so that the variability of the specified geometric parameters was well below the prescribed maximum tolerances, as follows. The vessel verticality and the horizontal positioning of its top lid in the square tank were determined with the digital protractor (having a precision of ±0.1 degrees) and were always 90.0° and 0.0°. The verticality of the shaft was similarly measured and was always 89.9–90.0° on one side of the shaft and 89.8–89.9° on a side perpendicular to that. Shaft centering in the vessel was obtained with the CenterChek™ device: the deviation from the perfect central position was measured along eight radial positions from the shaft to the vessel wall and it was always less than 1 division on the instrument dial (1 division = 1 mm). Furthermore, visual interpolation of the needle position indicated that the typical needle deflection was estimated to be no more than ~1/4 division. This would correspond to a variation in the vessel radius approximately equal to ±0.3 mm. The maximum shaft wobble was measured with the wobble meter (having a precision of ±0.01 mm) and was always less than 0.22 mm. Similarly, the maximum basket wobble at the lower edge of a (brand-new) basket was always less than 0.58 mm.

The basket was mounted on the shaft using the “basket grabber” and lowered into the glass vessel placed in the previously described custom-made plexiglass assembly. The basket was mounted at the central location within the vessel with a basket clearance off the vessel bottom of 25 mm, as specified in the USP, using the HeightChek™ tool. The square tank was filled with water so as to minimize the refractive effects at the curved surface of the glass vessel wall during PIV measurements. A 50-μm PSP stock suspension was separately prepared by adding the PSP to a solution of DI water and Tween 80 (about 2–3 droplets in 50-mL). This solution was stirred using a magnetic stirrer at 100 rpm for 5 min. The seeding particle suspension was added to DI water in a beaker and additional DI water was added until the desired volume of 900-mL was reached. The beaker content was then added to the dissolution vessel. The motor driving the shaft and basket rotated clockwise at 50, 75, or 100 rpm, corresponding to basket tip speeds of 0.067 m/s, 0.100 m/s, and 0.133 m/s and Reynolds numbers equal to 533, 800, and 1067, respectively.

**Fig. 8:**
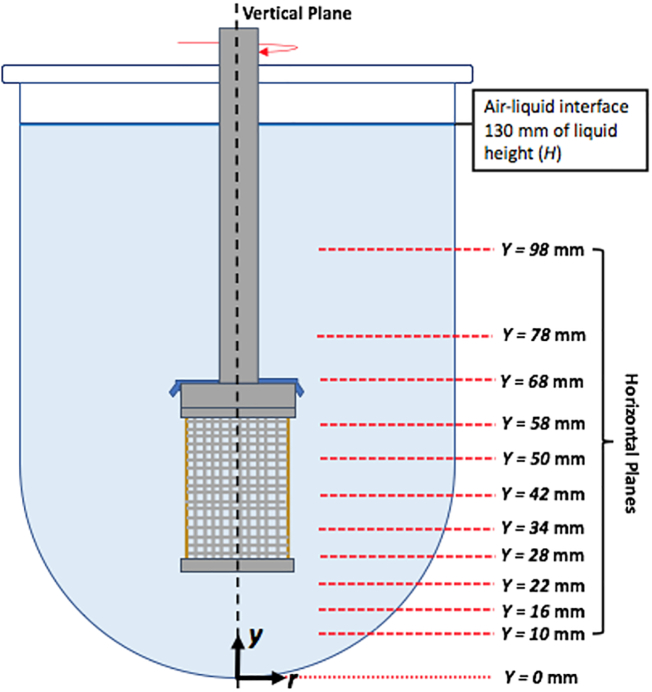
(a) Locations of the vertical cross section (vertical dash line) and horizontal cross sections (isosurfaces; horizontal red lines) where velocity distributions were obtained; (b) and (c) Laser-generated light sheets passing through the vertical and a representative horizontal cross section of the dissolution apparatus, respectively. (For interpretation of the references to colour in this figure legend, the reader is referred to the web version of this article.)

Experiments consisted of taking velocity measurements on a vertical cross section across the middle of the dissolution vessel, and on 11 horizontal cross sections at different isosurfaces in the vessel in order to quantify the velocity distribution of water inside the vessel, as shown in Fig. 8. The lowest point at the bottom of the dissolution vessel was defined as *Y* = 0 mm. The vessel was conceptually divided into three regions, i.e., above the basket, in the basket region (i.e., in the region of the vessel adjacent to the mesh bounded by the lower and upper edges of the basket), and below the basket. Three of the horizontal isosurfaces were located below the basket, i.e., at *Y* = 10 mm, *Y* = 16 mm, *Y* = 22 mm; five in the basket region, i.e., at *Y* *=* 28 mm; *Y* = 34 mm, *Y* = 42 mm, *Y* = 50 mm, and *Y* = 58 mm; and the other three above the basket, i.e., at *Y* = 68 mm, and *Y* = 78 mm, and *Y* = 98 mm.

When taking velocity measurements on the vertical cross section through the basket shaft, the double-pulsed laser was mounted in front of the dissolution vessel and traversed so that the resulting laser sheet with the thickness of 4 mm was centered on the basket shaft within a 0.25 mm level of precision. Measurements on vertical planes for all configurations were carried out first. Then, to take measurements on horizontal cross sections the camera was pointed toward the 45°mirror to capture the image reflected on the mirror, the laser head was turned by 90° to illuminate the vessel horizontally, and the laser was positioned at the correct elevation, as described above.

While working with the central vertical plane, the shaft and dissolution basket obstructed the laser beam penetrating from the opposite side of the vessel resulting in the shadow. For this reason, the velocity distribution on the vertical plane through the shaft centerline could only be obtained on the section of the dissolution vessel fully illuminated by the laser sheet, i.e., the entire cross section below the basket and half of the vessel at or above the basket. As for the measurements on the horizontal planes, i.e., with the laser generating a horizontal sheet and the camera viewing this cross section from the bottom of the vessel, velocity data could be collected on all sections below the basket. However, at or above the basket only the fraction of the cross section illuminated by the laser could be observed. This implies that the effects of any geometric asymmetry in the direction of the laser light could be obtained only in the region below the basket. However, the effects of geometric asymmetries perpendicular to the laser were still observable even in the basket region.

The time difference between two laser pulses, Δ*t,* was selected to be 30 ms, 20 ms and 10 ms at 50, 75, and 100 rpm, respectively, following the aforementioned procedure for the optimization of particle displacement. The laser light scattered by the particles was captured by the digital camera oriented perpendicularly with respect to the laser-light sheet and connected to the data acquisition system. PIV software (DynamicStudio 6.11.33 Software) was used to process the raw data captured by the camera and obtain the 2D velocity vectors on the selected plane. The software collected pairs of digitized images and subdivided them into small subregions (interrogation windows). In this work the size of the interrogation windows was typically 32 × 32 pixels. For each pair of images for a given interrogation window the background noise was subtracted to remove most of the bright areas caused by reflections from the glass vessel and then analyzed using adaptive PIV to determine the spatial displacement of the particles within the illuminated plane. The average velocity distribution convergence was obtained to determine statistically independent PIV data from the image pairs in order to generate consistent and reliable velocity data for each interrogation window. For each x-y point associated with each interrogation window the PIV software then generated data files of the 2D x-y cartesian components of the average velocities on the plane on which the PIV images were taken. The software additionally calculated the resulting velocity vectors on that plane, which could be then plotted to give 2D velocity vector plots or contour plots on the cross section of interest (vertical or horizontal). The PIV velocity distribution results were then post-processed using Tecplot® (360 EX 2018 R2) program to generate velocity vector and contour images.

After a number of preliminary experiments, the optimal number of PIV image pairs was found. As a result, in all experiments 1200 image pairs were collected to produce 1200 instantaneous velocity data points for each interrogation window and obtain the velocity distribution for each agitation speed on each cross section.

## 3 Results

### 3.1 Results of preliminary experiments

**Fig. 9:**
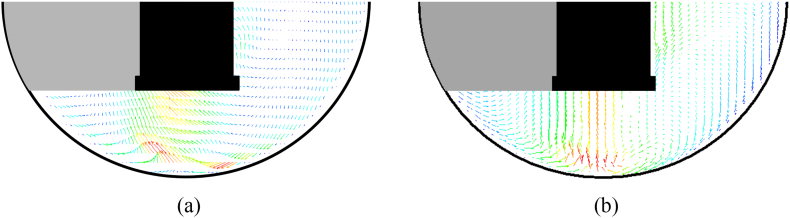
Example of velocity vectors on a vertical cross-section in the lower portion of Apparatus 1 using: (a) standard calibration and alignment procedures; and (b) improved calibration and alignment procedures.

A significant number of preliminary experiments were conducted prior to collecting actual data as a consequence of the extreme sensitivity of the system to a variety of factors. Initially, PIV experiments were conducted using “standard calibration” procedures on the USP Apparatus 1 system and the Distek™ apparatus, such as proper horizontal positioning of the equipment (via the leveling bubble built into the apparatus) or measurement of the extent of basket wobbling using a wobble meter, as described in the Experimental Section. In all cases, the variability in these parameters was within the USP specifications (Table 2). Similarly, the vertical laser sheet produced by the PIV system was properly aligned with the vessel centerline as routinely done for other systems but without using any special precautions. However, after obtaining a long string of apparently varying results for the velocity, it became apparent that the system was very sensitive to even minor variations in the geometry of the system or the alignment of the PIV components, and that special precautions needed to be taken. As a result, the system was re-calibrated using a new basket and a new set of calibration devices and applying even more stringent calibration requirements than those used previously (“improved calibration”). To show differences in velocity vectors obtained by using the two calibration procedures more clearly, the vectors on a vertical cross section through the vessel centerline are presented in Fig. 9. Vectors obtained first by properly calibrating the system and properly aligning the laser but without taking any special precautions about “improved calibration” of the different components (Fig. 9a) and then after implementing the “improved calibration” protocol (Fig. 9b) are shown. One can see from this figure that there is more pronounced lack of symmetry in the velocity profiles obtained before careful calibration (Fig. 9a) than when all system components (including both the dissolution apparatus assembly and the all PIV components, and especially the laser) were more carefully calibrated or positioned (Fig. 9b). However, this figure also shows that even when this improved calibration procedure was in place there was still some degree of asymmetry in the velocity profiles. This improved calibration procedure was kept in place in all successive experiments presented in the remainder of this section.

While the different calibration procedures discussed above might not necessarily result in variability in dissolution results, improved calibration was still required to ensure repeatable images that could result in averaged symmetric vector plots for the PIV data. However, it should be noted that in the industrial practice it is unlikely that the same degree of consistency used here could be achieved. For example, baskets are typically reused and are not always new, and it is unlikely that the typical operator handles the baskets with the same level of caution as we did in this work after we realized the system's sensitivity to even minute deviations from symmetry.

### 3.2 Velocity vector plots and velocity contour plots

**Fig. 10:**
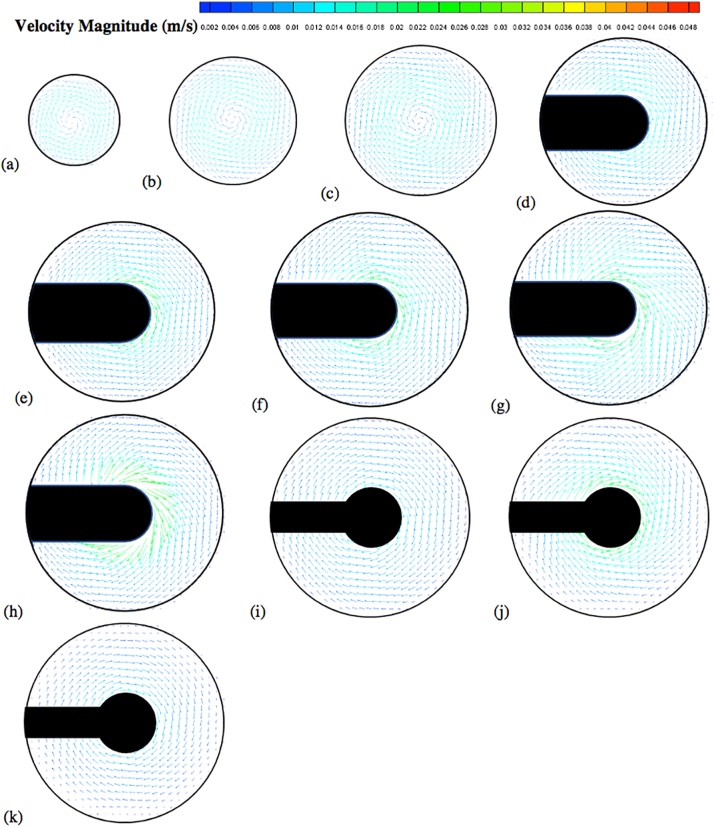
Velocity vector colored by velocity magnitude (m/s) on different horizontal planes (isosurfaces) for *V* = 900 mL and *N* = 50 rpm; isosurfaces at (a) *Y* = 10 mm; (b) *Y* = 16 mm; (c) *Y* = 22 mm; (d) *Y* = 28 mm; (e) *Y* = 34 mm; (f) *Y* = 42 mm; (g) *Y* = 50 mm; (h) *Y* = 58 mm; (i) *Y* = 68 mm; (j) *Y* = 78 mm; (k) *Y* = 98 mm.

**Fig. 11:**
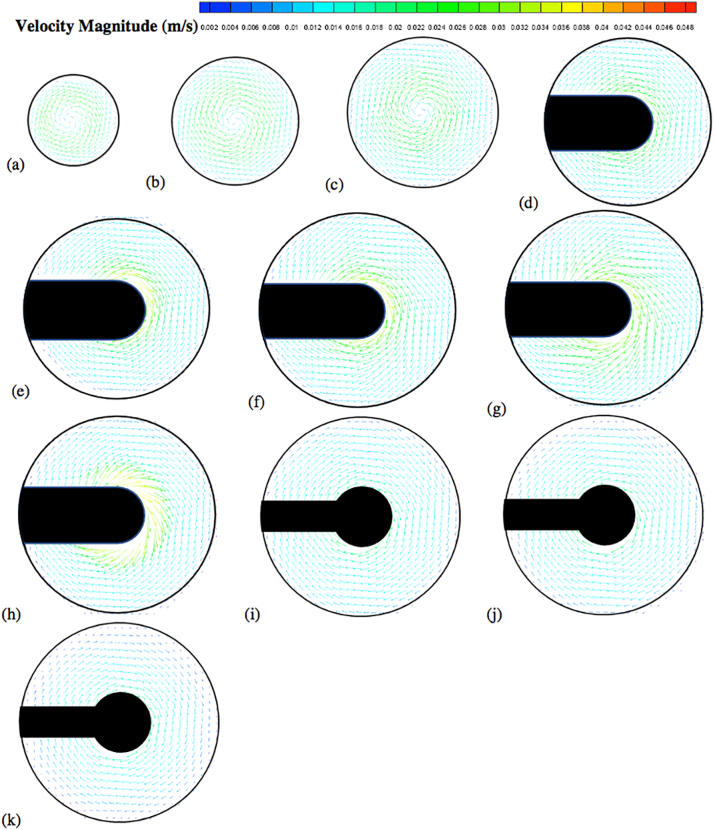
Velocity vector colored by velocity magnitude (m/s) on different horizontal planes (isosurfaces) for *V* = 900 mL and *N* = 75 rpm; isosurfaces at (a) *Y* = 10 mm; (b) *Y* = 16 mm; (c) *Y* = 22 mm; (d) *Y* = 28 mm; (e) *Y* = 34 mm; (f) *Y* = 42 mm; (g) *Y* = 50 mm; (h) *Y* = 58 mm; (i) *Y* = 68 mm; (j) *Y* = 78 mm; (k) *Y* = 98 mm.

**Fig. 12:**
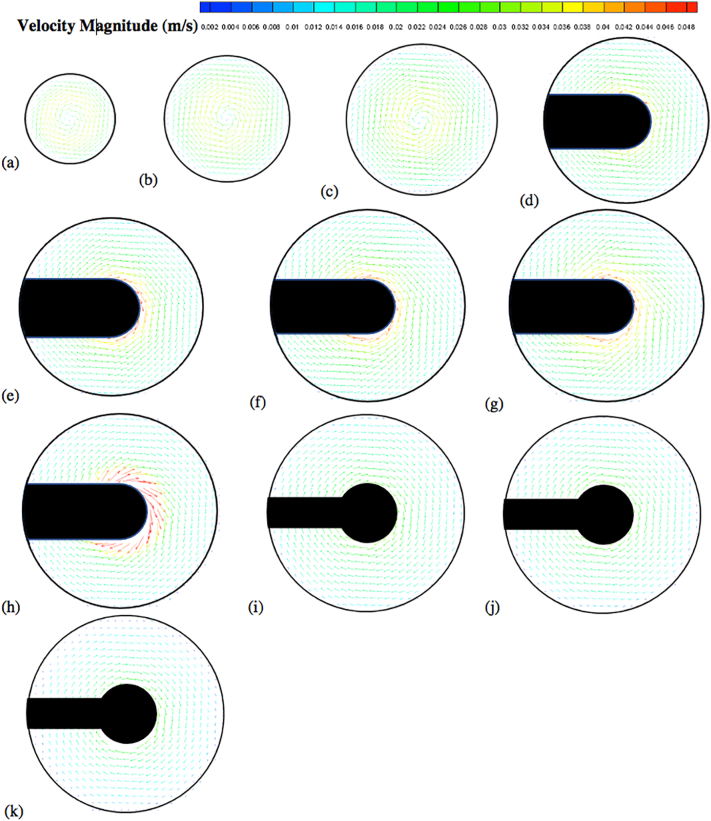
Velocity vectors colored by velocity magnitude (m/s) on different horizontal planes (isosurfaces) for *V* = 900 mL and *N* = 100 rpm; isosurfaces at (a) *Y* = 10 mm; (b) *Y* = 16 mm; (c) *Y* = 22 mm; (d) *Y* = 28 mm; (e) *Y* = 34 mm; (f) *Y* = 42 mm; (g) *Y* = 50 mm; (h) *Y* = 58 mm; (i) *Y* = 68 mm; (j) *Y* = 78 mm; (k) *Y* = 98 mm.

**Fig. 13:**
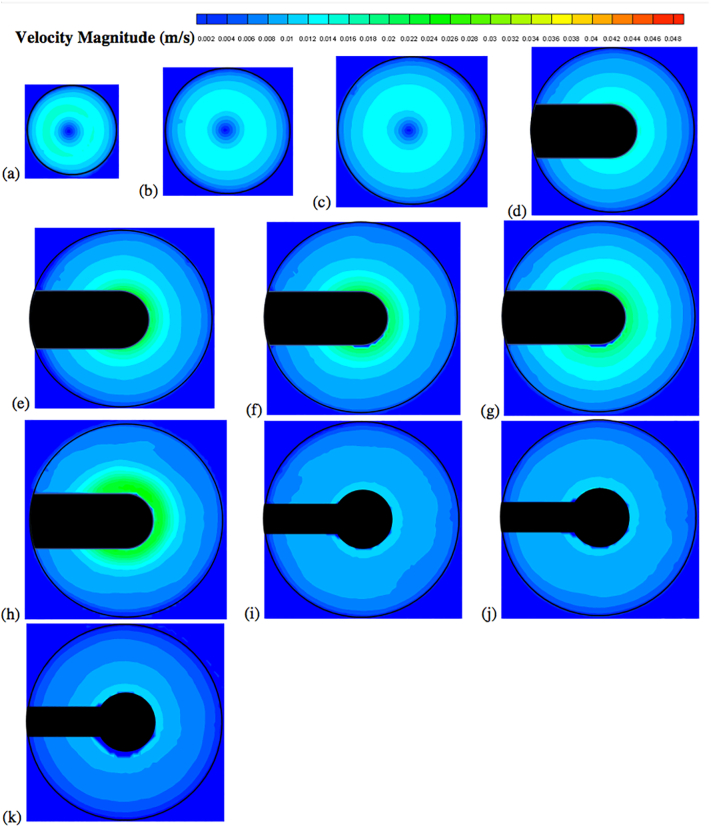
Velocity contour plots colored by velocity magnitude (m/s) on different horizontal planes (isosurfaces) for *V* = 900 mL and *N* = 50 rpm; isosurfaces at (a) *Y* = 10 mm; (b) *Y* = 16 mm; (c) *Y* = 22 mm; (d) *Y* = 28 mm; (e) *Y* = 34 mm; (f) *Y* = 42 mm; (g) *Y* = 50 mm; (h) *Y* = 58 mm; (i) *Y* = 68 mm; (j) *Y* = 78 mm; (k) *Y* = 98 mm.

**Fig. 14:**
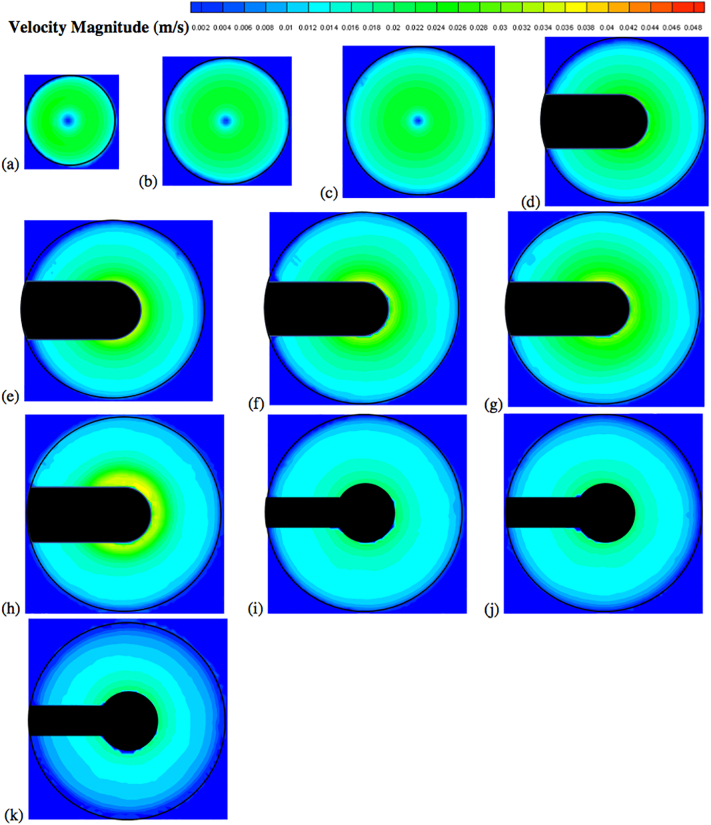
Velocity contour plots colored by velocity magnitude (m/s) on different horizontal planes (isosurfaces) for *V* = 900 mL and *N* = 75 rpm; isosurfaces at (a) *Y* = 10 mm; (b) *Y* = 16 mm; (c) *Y* = 22 mm; (d) *Y* = 28 mm; (e) *Y* = 34 mm; (f) *Y* = 42 mm; (g) *Y* = 50 mm; (h) *Y* = 58 mm; (i) *Y* = 68 mm; (j) *Y* = 78 mm; (k) *Y* = 98 mm.

**Fig. 15:**
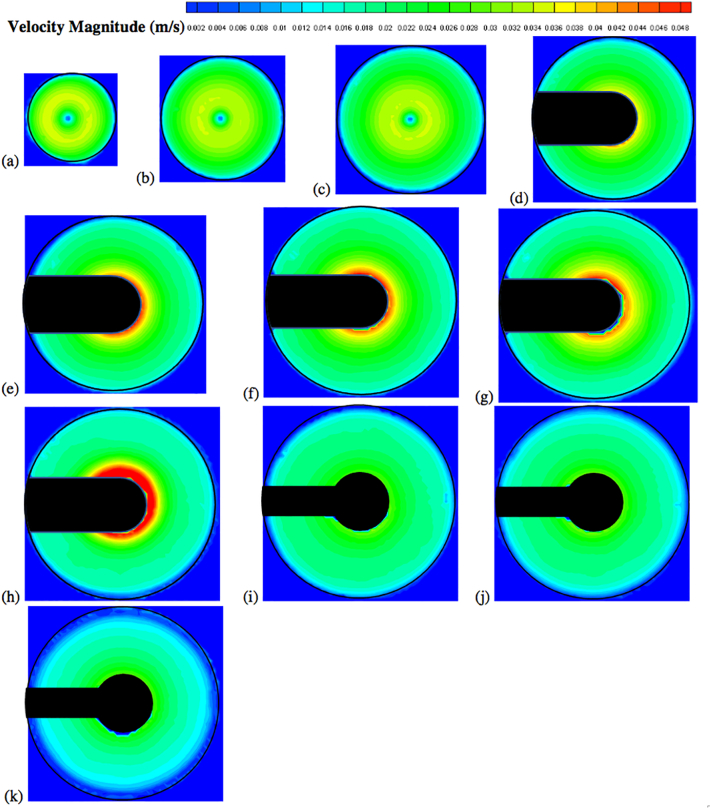
Velocity contour plots colored by velocity magnitude (m/s) on different horizontal planes (isosurfaces) for *V* = 900 mL and *N* = 100 rpm; isosurfaces at (a) *Y* = 10 mm; (b) *Y* = 16 mm; (c) *Y* = 22 mm; (d) *Y* = 28 mm; (e) *Y* = 34 mm; (f) *Y* = 42 mm; (g) *Y* = 50 mm; (h) *Y* = 58 mm; (i) *Y* = 68 mm; (j) *Y* = 78 mm; (k) *Y* = 98 mm.

**Fig. 16:**
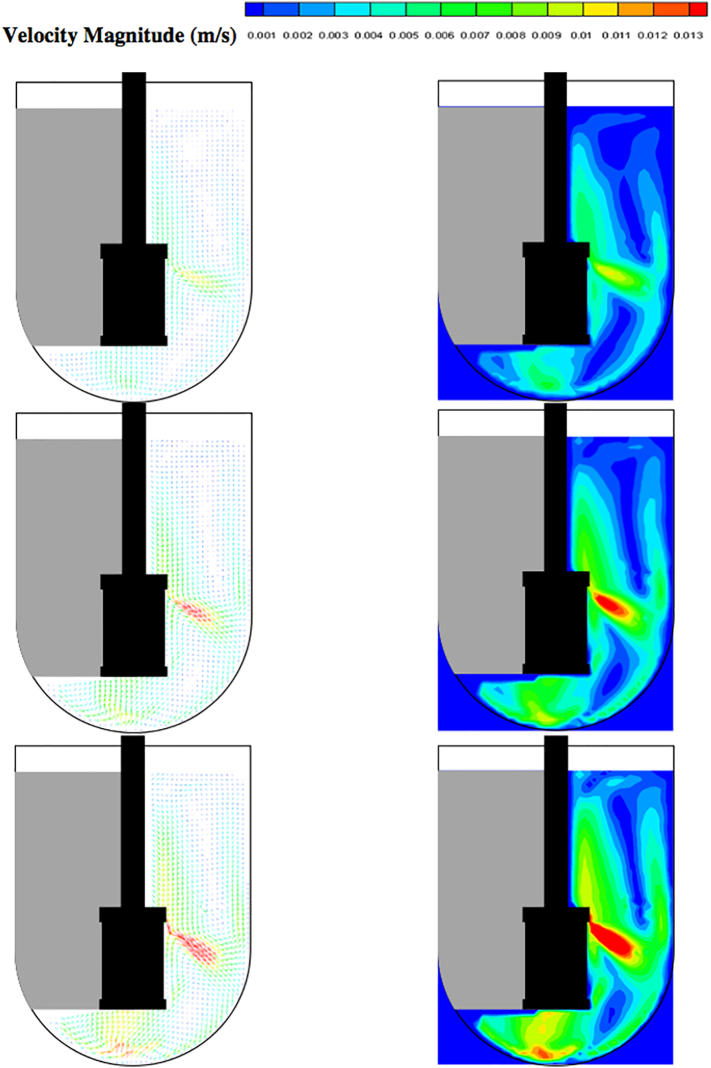
Velocity vectors (left panels) and velocity contour plots (right panels) colored by velocity magnitude (m/s) at different agitation speeds for *V* = 900 mL; *N* = 50 rpm (top panels); *N* = 75 rpm (middle panels); *N* = 100 rpm (bottom panels).

The velocity vector and contour plots are presented in Fig. 10, Fig. 11, Fig. 12, Fig. 13, Fig. 14, Fig. 15, Fig. 16. The different colors in these figures indicate different velocity magnitudes, and the gray or black regions indicate regions that could not be investigated with the PIV system since some components of the apparatus (basket, shaft) blocked either the laser sheet or the camera view. This is evident in the figures reporting the velocities on the vertical cross section, where the basket or the shaft blocked the laser sheet in most of the left side of the vessel. As for the velocities on the horizontal cross sections, the basket intercepted the laser sheet on the isosurfaces at *Y* = 28 mm, 34 mm, 42 mm, 50 mm, and 58 mm, and the shaft on the isosurfaces at 68 mm, 78 mm and 98 mm.

Fig. 10 shows the experimental velocity vectors from PIV measurements on different horizontal cross sections (isosurfaces) in the vessel for *N* = 50 rpm. Similar figures are presented for *N* = 75 rpm (Fig. 11) and *N* = 100 rpm (Fig. 12). Fig. 13, Fig. 14, Fig. 15 show the corresponding velocity contour plots for the same three agitation speeds, respectively. In general, the flow in the whole vessel was dominated by the tangential velocity component resulting from rotation of the basket, and especially in the region surrounding the basket. The velocities typically increased with agitation speed and were highest in the region immediately adjacent to the basket. Interestingly, the velocities in the inner central region below the basket, where settling tablet fragments are typically located during a dissolution process after a tablet disintegrates, were still very low, although the velocities in this central zone increased with increasing agitation speed. This can be clearly seen by looking at the velocities on the low-*Y* planes (panels (a), (b) and (c)). Despite all the precautions taken to ensure that the system was properly assembled and calibrated, some small asymmetries in the flow could still be observed on some horizontal planes, especially below the basket and at higher agitation speeds. The contour plots (Fig. 13, Fig. 14, Fig. 15) evidence even more clearly that the flow was not perfectly symmetrical, as indicated by the slight off-center position of the center-of-rotation for the planes below the basket, and the minor variations in the velocity contours with angular position on the planes at the basket level (panels (d)-(h)).

Fig. 16 shows the axial and radial velocity vectors and velocity contour plots on a vertical cross section through the vessel centerline at all three agitation speeds. The first point to notice is the extremely weak axial and radial velocities, which were always below about 10 mm/s even at 100 rpm [Remark: the velocity scale in all the panels of this figure is expanded with respect to the scale in the corresponding figures on the horizontal planes]. Such low velocities are to be expected in this system since the basket is not similar to an impeller capable of radially pumping the liquid. When the agitation speed was 50 rpm the largest magnitude of the axial and radial velocity observable on the vertical plane was on the order of 5 mm/s, but most of the velocities were much smaller. Even at 100 rpm these velocities were still very low. Fig. 16 also shows that the flow was not symmetric, which can be expected given that the velocities are so weak that any minute deviation from perfect symmetry can have an appreciable impact, despite all the precautions taken in this work. A small jet could be seen emanating radially near the top of the basket and increasing in intensity with increasing agitation speed. This flow is likely the result of the presence of the three-small clips holding the basket (see Fig. 5), which act as miniscule “impeller blades.” The small radial flow generated by an odd number of clips would likely contribute to create small and possibly spatially periodic flow instabilities which would then propagate throughout the vessel further contributing to breaking symmetry in a system already very gently stirred and dominated by the weak but, relatively speaking, much stronger tangential flow generated by the basket.

The flow features observable in Fig. 16, such as the radial jet and the small flow asymmetry, could be clearly observed on the vertical plane, but, in general they had a negligible impact on the flow on the horizontal planes (Fig. 10, Fig. 11, Fig. 12, Fig. 13, Fig. 14, Fig. 15) since the radial velocities were typically much smaller (about one order of magnitude or more) than the tangential velocities. However, the effect of the radial velocity was observable on one of the horizontal planes, i.e., that at *Y* = 58 mm (all panels (h) in Fig. 10, Fig. 11, Fig. 12, Fig. 13, Fig. 14, Fig. 15) since on this plane the radial jet of Fig. 16 produced stronger radial velocities than on the other horizontal planes. In the jet area the radial velocities, while still weaker than the corresponding tangential velocities on the same plane, were clearly larger than the radial velocities anywhere else, peaking to about 1/3 to 1/2 of the tangential velocity magnitude, depending on the agitation speed. This radial component contributed to a slight increase in the magnitude of the resulting vectors on this plane and to slightly change the direction of those vectors, which was otherwise mostly tangential. This effect can be seen as an expanded higher velocity region surrounding the basket on this plane, compared to the same region on the other horizontal planes in the same figures. For example, a close examination of the point-by-point radial velocities in this region (actual detailed data not presented here) showed that at a radial distance equal to about 18 mm from the shaft center (*r/R* ≅ 0.36, i.e., just 5–6 mm away from the cylindrical basket mesh), the radial velocity was only 5–6% of the tangential velocity for *Y* = 42 mm and *Y* = 50 mm (panels (f) and (g)), but for *Y* = 58 mm (panels (h)) the radial velocity was 35–47% of the tangential velocity, depending on the agitation speed. The radial flow effect on this plane rapidly vanished for larger *r/R* values. This slightly increased flow rate near the upper end of the basket could possibly enhance the flow in the vicinity of a floating solid dosage form, which is likely to reside in the upper region of the basket, since the basket is also designed to assist with submersing floating dosage forms. Fig. 16 additionally shows that below the basket there was a weak upward flow penetrating the bottom mesh of the basket. This flow is critical for the performance of any dissolution test conducted in USP Apparatus 1 since, because of its mainly axial direction, this flow impinges directly on the tablet within the basket. Therefore, any variation in the basket agitation speed will, in turn, affect the intensity of the flow entering the lower mesh of basket, which possibly will directly affect the mass transfer coefficient and therefore the mass transfer rate between the tablet and the dissolution medium, with obvious implication for the rate of tablet dissolution and, possibly, the suspension of the tablet off the basket bottom within the basket. These aspects are examined below in greater detail through the analysis of the velocity profiles on specific isosurfaces and especially the isosurface immediately below the basket.

### 3.3 Detailed comparison of the nondimensional velocity profiles on specific iso-surfaces

The previous figures illustrate quite clearly the change in the magnitude of the velocity vectors as a function of agitation speed and location within the vessel, but they are difficult to use for quantitative comparison of the effect of agitation speed on the velocity profiles. Therefore, some of the same velocity data are presented here as graphs of the nondimensional velocities in a given direction as a function of the nondimensional radial coordinate, for selected isosurfaces. The nondimensional velocities are defined here as *U*_*a*_/*U*_*tip*_ (where *U*_*a*_ is the velocity in the axial (vertical) direction and *U*_*tip*_ is the basket tip velocity calculated at a radial distance equal to radius of the basket bottom edge), *U*_*r*_/*U*_*tip*_ (where *U*_*r*_ is the velocity in the radial direction), and *U*_*t*_/*U*_*tip*_ (where *U*_*t*_ is the velocity in the tangential direction). The velocities were considered positive if they are oriented upward for *U*_*a*_, outwards from the vessel centerline for *U*_*r*_, and counterclockwise for *U*_*t*_, respectively. The nondimensional radial coordinate is defined as *r/R* coordinate, where *r* is the radial coordinate (with *r* = 0 at the vertical vessel centerline) and *R* is the radius of the dissolution vessel, as shown in Fig. 8.

**Fig. 17:**
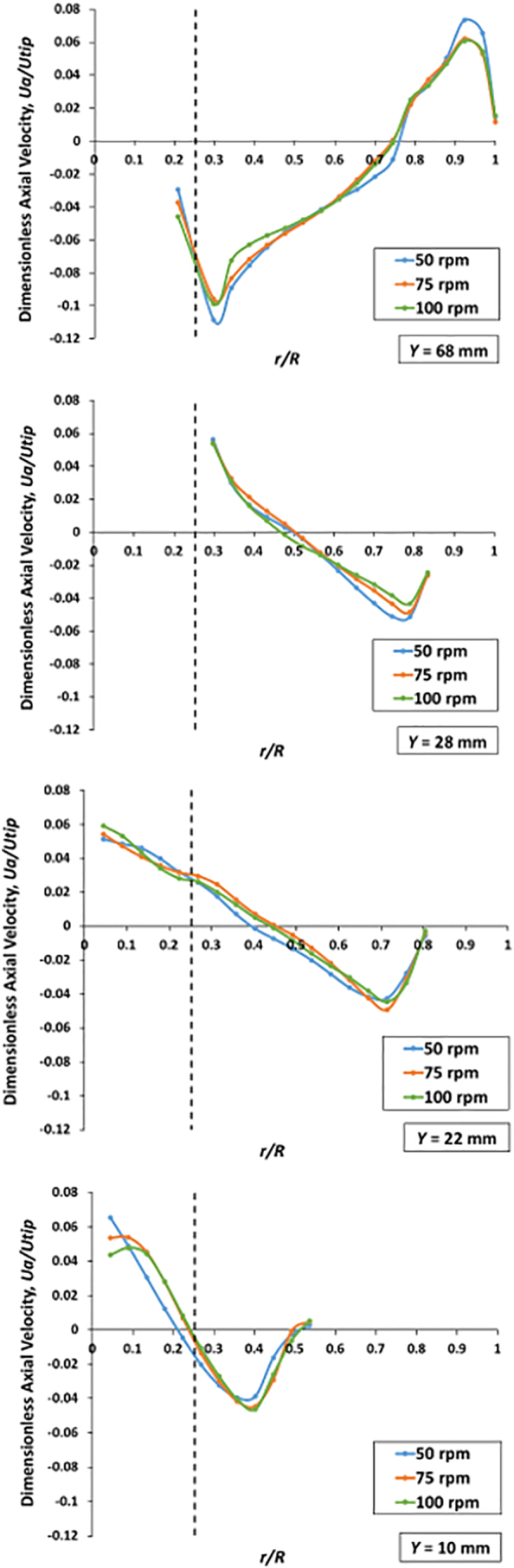
Nondimensional axial velocities *U*_*a*_*/U*_*tip*_ as a function of the nondimensional radial coordinate *r/R* on isosurfaces at *Y* = 10 mm and 22 mm (below the basket), 28 mm (in the basket region), and 68 mm (above the basket) and agitation speeds of 50, 75, and 100 rpm. *V* = 900 mL.

**Fig. 18:**
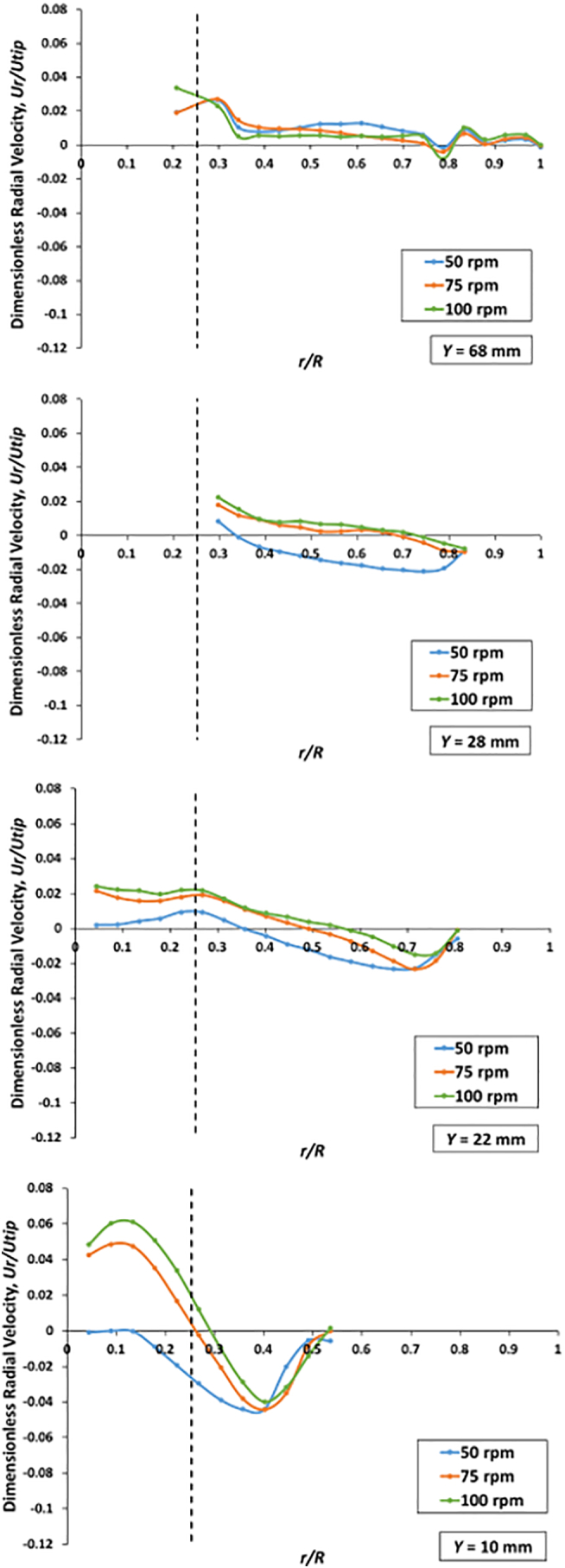
Nondimensional radial velocities *U*_*r*_*/U*_*tip*_ as a function of the nondimensional radial coordinate *r/R* on isosurfaces at *Y* = 10 mm and 22 mm (below the basket), 28 mm (in the basket region), and 68 mm (above the basket) and agitation speeds of 50, 75, and 100 rpm; *V* = 900 mL.

**Fig. 19:**
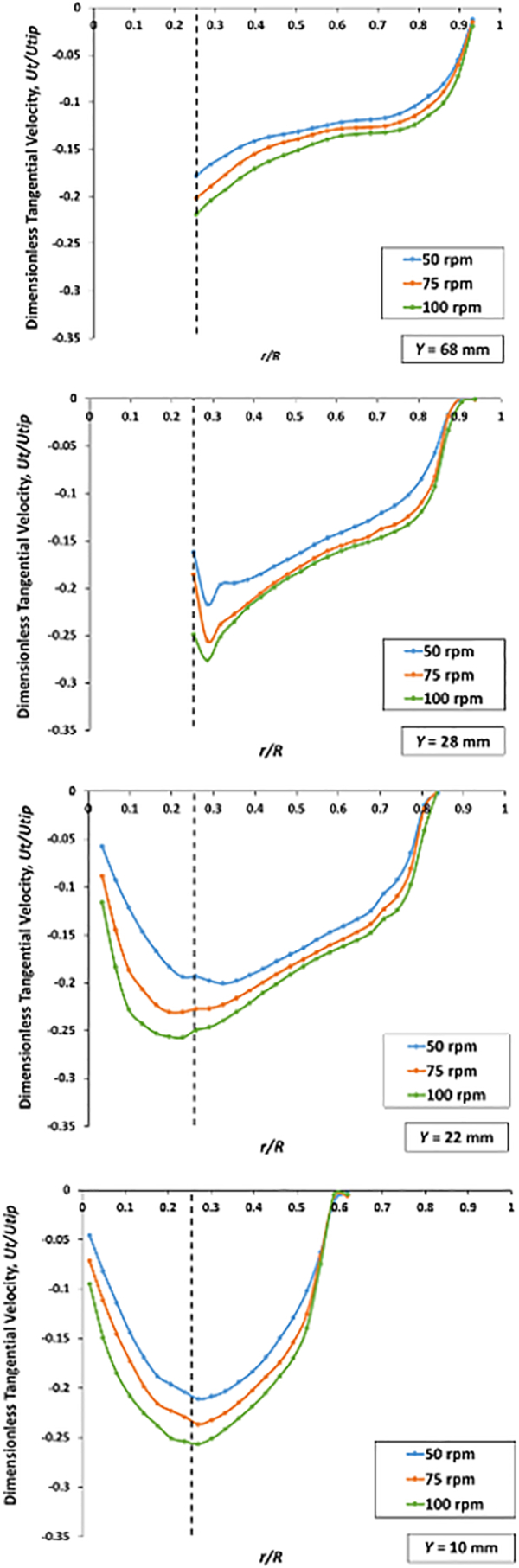
Nondimensional tangential velocities *U*_*t*_*/U*_*tip*_ as a function of the nondimensional radial coordinate *r/R* on isosurfaces at *Y* = 10 mm and 22 mm (below the basket), 28 mm (in the basket region), and 68 mm (above the basket) and agitation speeds of 50, 75, and 100 rpm; *V* = 900 mL.

Fig. 17, Fig. 18, Fig. 19 show graphs of the nondimensional axial, radial and tangential velocities, respectively, at three different agitation speeds and for four levels, i.e., on the isosurfaces at *Y* = 10 mm (below the basket), *Y* = 22 mm (just below the basket), *Y* = 28 mm (low within the basket region, near where the tablet usually rests within the basket), and *Y* = 68 mm (above the basket, near the three small clips). It should be noticed that the dashed line in these figures indicate the edge of the basket and that the axes in these figures have different scales depending on the velocity magnitude of each velocity component since the tangential velocity components were typically one or two orders of magnitude larger than the axial and radial components.

#### 3.3.1 Nondimensional velocity profiles *below* the basket

In the regions below the basket (*Y* *=* 10 mm and *Y* *=* 22 mm) shown in Fig. 17, one can see that the nondimensional axial velocity profiles were nearly identical to each other, which means that the axial velocities generally scaled quite well with the agitation speed in this region. The only exception the region very close to the center of the vessel (*r/R* < ~0.1) on the lowest level (*Y* = 10 mm), where the actual axial velocities *U*_*a*_'s appeared to be lower than the velocities in other region of the vessel, and nearly identical and independent of agitation speed (since *U*_*a*_ = (*U*_*a*_*/U*_*tip*_) × *U*_*tip*_). This region is important since the plane at *Y* = 10 mm is very close to the bottom of the dissolution vessel where tablet fragments falling off the basket are typically found. However, everywhere else in the region directly below the basket (*r/R* < ~0.25) the non-dimensional velocities were largely independent of the agitation speed. This is an important observation in general, but it is especially relevant for the isosurface at *Y* = 22 mm since this isosurface is immediately below the basket, which implies that the flow generated by the velocities on this plane directly impacts the flow *through* the basket.. Both these aspects are more extensively examined in the Discussion section below.

Fig. 18 for the same *Y*-isosurfaces shows the plot for the nondimensional radial velocity. Here the curves at both levels but different agitation speeds appeared to have similar shapes and trends but different values. For *Y* = 10 mm, the profiles in the outer region were similarly shaped but less so in the core region below the basket. However, Fig. 16 shows that the flow near the vessel bottom was slightly non-symmetrical, and this (minor) lack of symmetry is reflected in the radial velocities in Fig. 18. Furthermore, the radial velocities on this plane (bottom panel in Fig. 18) were all exceedingly low, i.e., less than about 6% of the basket tip speed at all agitation speeds. This corresponds to less than 7 mm/s for the 100-rpm case and about 2.5 mm/s at 50 rpm. However, for most *r/R* values, the radial velocities were much smaller and on the order of 1 mm/s or less.

Finally, Fig. 19 shows the nondimensional tangential velocity profiles (the scale is this figure is different from the previous two). The graphs show that the tangential velocities, unlike the radial and axial velocities, were on the same order of magnitude as the tip velocity, with peak values of about 25% of *U*_*tip*_ at 100 rpm, indicating that the tangential component of the velocity dominated the flow field throughout the vessel. The similarity of the profiles in each panel indicates that the tangential velocity scaled well with the agitation speed. The greatest deviation from this behavior was for *Y* = 22 mm, showing that the three curves were more differentiated. However, the velocity decayed rapidly with the radial distance in a manner similar to that appearing in irrotational vortices (where *U*_*t*_ is proportional to 1/*r*). Fig. 19 seems to indicate that the tangential component of the velocity was approximately similar to that of two-dimensional Rankine vortex, with a central core (where observable, as for *Y* = 10 mm) rotating as a solid body, for *r/R* < ~0.25, and an outer region, for ~0.25 < *r*/*R* < ~0.9, where the vortex was approximately irrotational. Near the wall (*r/R* > ~0.9) the velocity dropped rapidly with radial distance to become zero at the wall. The small velocity increase near the vessel wall was likely an artifact caused by the curvature of the glass and the reflection of laser light off the glass generating a small false velocity reading.

More in general, a comparison of Fig. 17, Fig. 18, Fig. 19 shows, again, that the dominant velocity component was the tangential component on all horizontal planes and that the radial and axial components were about one order of magnitude smaller than the tangential component.

#### 3.3.2 Nondimensional velocity profiles *in* the basket region

Fig. 17, Fig. 18 (second panels from top) show, respectively, the nondimensional axial and radial velocity profiles on the isosurfaces in the basket region (at *Y* *=* 28 mm, i.e., low within the basket region, near where the tablet usually rests within the basket). The axial and radial velocities in the vessel scaled up quite well with agitation speed. The axial components were generally weak and positive, i.e., generating an upward flow near the basket, while the flows became negative, implying a downward flow, near the vessel wall. The graph shows that the peak axial velocity was about 6% of *U*_*tip*_. The non-dimensional radial components were much weaker than the axial components. Fig. 19, at the same elevation, shows the nondimensional tangential velocity profiles. The similarity of the profiles between a level below the basket (*Y* *=* 22 mm and *r/R* *>* *~*0.25) and this level (*Y* *=* 28 mm) can also be observed. The tangential velocity scaled up well with agitation speed. The graphs indicated that the tangential velocities were on the same order of magnitude as the tip velocity, with peak values of about 25–30% of *U*_*tip*_ at 100 rpm.

#### 3.3.3 Nondimensional velocity profiles *above* the basket

Fig. 17 (top panel) shows the nondimensional axial velocity profiles on the isosurface above the basket (*Y* *=* 68 mm). The non-dimensional axial velocities also scale quite well with the agitation speed in this region since the profiles are similarly shaped. Fig. 18 (top panel) shows the nondimensional radial velocity on the isosurface at *Y* *=* 68 mm. The radial velocities in a region adjacent to the basket (at *r/R* = ~0.25) were the highest and then gradually decreased toward the vessel wall. Overall, the radial velocities on this isosurface were greater than those on the other *Y-*isosurfaces shown in this figure. This is likely caused by the small jet radiating from the three-small clips holding the basket, which act as minuscule “impeller blades.” Fig. 19 (top panel) shows the nondimensional tangential velocity profiles. Again, the tangential velocities, unlike the radial and axial velocities, were on the same order of magnitude as the tip velocity, with peak values of about 23% of *U*_*tip*_ at 100 rpm. The flow was dominated by the tangential components and scaled well with the basket tip speed. The tangential velocities were additionally found to be slightly weaker than that of other *Y-*isosurfaces because this elevation is further away from the basket.

## 4 Discussion

This study is possibly the first study aimed at fully characterizing and quantifying the velocity distribution experimentally inside a USP Apparatus 1 for the whole dissolution vessel. In general, we found that small deviations from perfect geometrical symmetry resulted in asymmetries in all velocity components. This seems to be a common feature of this apparatus, irrespective of how carefully the system is set up. In other terms, any small imperfection or asymmetry in any component of the system, and especially the basket, can shift the flow off the vessel centerline even if the deviations are minute or within commonly accepted tolerance ranges. Deviations from perfect symmetry are even more likely to occur in the industrial practice as a result of small geometrical misalignments of the system components (although within established tolerances) and the wear and tear of the equipment, such as small deformations of the basket mesh while handling it and mounting it on the shaft, or slight bending of the shaft.

As seen in all agitated unbaffled systems, the velocity distribution was strongly dominated by the tangential velocity component on all horizontal cross-sections in the PIV measurements. In general, the tangential velocities scaled quite well with agitation speed, but less so in the region below the basket. The axial and radial velocities generally increased with agitation speed, the only exception being the central region near the bottom of the vessel and with a diameter roughly equal to that of the basket where these velocity components were somewhat independent of agitation speed. This implies that drug fragments dropping out of the basket upon disintegration and residing at the bottom of the vessel would be exposed to similar radial and axial velocities (but not tangential velocities) at all agitation speeds, promoting coning at the center of the vessel bottom which could affect the mass transfer rate of the tablet fragment and hence the drug dissolution profiles. On the other hand, and as already mentioned, increasing the agitation speed also resulted in an increase in the tangential velocities below the basket, which, combined with the small (and typically asymmetric) radial velocities in this region could help transport the solids fragments out of the coning region below the basket and promote breaking up the cone.

An especially relevant aspect of the flow in Apparatus 1 is that associated with the axial velocities within the region for *r/R* < ~0.25 on the plane at *Y* = 22 mm, which lies just below the basket (Fig. 17). These velocities produce a flow which is directly pointing upward toward the horizontal bottom mesh of the basket. Because of the proximity of the plane at *Y* = 22 mm with the bottom of the basket at *Y* = 25 mm, this flow can only directly impinge on the bottom mesh of the basket and fully penetrate the basket, as one can also visually observe in Fig. 16. In addition, Fig. 17 shows that the non-dimensional velocity profiles, *U*_*a*_*/U*_*tip*_, at *Y* = 22 mm at different agitation speeds for *r/R* < 0.25 are nearly overlapping. Since *U*_*tip*_ is directly proportional to the basket agitation speed (*U*_*tip*_ = *π×N×ODB*, with *N* in revolutions per second (rps)) this implies that *U*_*a*_ is directly proportional to *U*_*tip*_, which additionally indicates that *the velocities of the fluid entering through the bottom mesh of the basket, and hence the flow penetrating the basket, increase in directly proportionality to the agitation speed*. This implies that increasing the agitation speed would increase this flow and therefore also possibly increase the tablet-dissolution medium mass transfer rate, thus promoting tablet dissolution. Furthermore, a higher flow rate through the basket would entail that the tablet could be more easily suspended within the basket as the agitation speed is increased. These are major conclusions of our work since they can provide some guidance to the dissolution practitioner on the effect of agitation speed on the flow within the basket. It is interesting to notice that although the velocity profiles inside the basket itself could not be directly investigated with the PIV system because of the non-transparency of the basket, the corresponding flow features immediately next to the basket, including the velocities through the bottom mesh, could be measured.

Fig. 16 shows that the basket rotation generated two recirculation loops, demarcated by the angled radial jet near the top of the basket, as clearly shown in the velocity vectors on the vertical cross-section. The first and stronger loop affected the lower portion of the vessel forming a flow that moved downwards along the vessel wall, converged toward the center of the vessel below the basket, moved centrally upward to enter the bottom end of the basket, and closed the loop near the top edge of the basket. The second weaker loop contributed to the upper recirculation above the basket, generating a flow that moved upwards along the vessel wall, then turned radially inwards along the liquid-air interface converging toward the top center of the liquid, and finally rejoined the jet to close the loop. Clearly the fact that the basket is not an effective pumping device implies that the upper flow it generates cannot strongly extend too far away above the basket.

Interestingly, the flow patterns in Apparatus 1 present significant similarities to those in Apparatus 2, despite the obvious difference in the primary moving device (basket vs. paddle). A quantitative comparison can be made using the results of this work and the detailed experimental velocity data of previous investigations with Apparatus 2 (Bai et al., 2007; Bai et al., 2011). In both systems the main flow is strongly tangential, which is typical of all unbaffled systems, with limited axial and radial components, and in both cases, secondary flows forming recirculation loops can be observed. Somewhat unexpectedly, the *non-dimensional* tangential velocities in Apparatus 2, although much larger than in Apparatus 1, were found to be of the same order of magnitude in most regions of the vessel (~0.4–0.5 for Apparatus 2 vs. ~0.2–0.3 for Apparatus 1) despite the fact that Apparatus 1 is not stirred by a “real” impeller. The non-dimensional tangential velocity profiles decreased more rapidly in Apparatus 1 than in Apparatus 2, i.e., the tangential motion imparted by the basket to the fluid did not extend as strongly with distance from the shaft as that exerted by the paddle. However, it should be remarked that the actual *dimensional* velocities in Apparatus 1 and Apparatus 2 are very different because of the difference in tip speed resulting from the much smaller diameter of the basket vs. that of the paddle, i.e., 25.46 mm vs. 74.1 mm, respectively (recalling that *U*_*tip*_ is directly proportional to the impeller diameter).

Also, both apparatuses are poor axial (vertical) mixing devices, as one can see by comparing the non-dimensional axial velocities which were similar in absolute peak magnitude (~0.1) in both systems, and even worse radial mixers, since the non-dimensional radial velocity was typically even smaller in both apparatuses, except in the vicinity of the paddle for Apparatus 2. The two systems also partially share another common feature, in that the central core region between the bottom of the vessel and the basket (Apparatus 1) or the lower edge of the impeller (Apparatus 2) is characterized by overall small velocities especially in the radial and axial directions. In fact, in both systems similar coning effects can be observed at low stirring speeds when a tablet disintegrates rapidly during the dissolution test and, in the case of Apparatus 1, the granules are small enough to escape the rotating basket. The resulting granules may accumulate at the vessel bottom forming a cone of loosely aggregating particles under the basket or impeller (Bai and Armenante, 2009; Higuchi et al., 2014; Todaro et al., 2017). Finally, both systems appear to be very sensitive to small asymmetries, as it was observed in Apparatus 2 when the paddle was placed in a slightly asymmetric position (but still within the USP specifications) (Bai and Armenante, 2008). Small inserts, such as fiber optic probes, may also introduce flow asymmetries, as evidenced by changes in dissolution results for Apparatus 2 (Wang et al., 2013; Zhang et al., 2013). Even though such inserts were not considered in this study, it is logical to assume that, if used, they would affect the hydrodynamics in Apparatus 1 as well.

The results found in this work for Apparatus 1 can be compared with the limited CFD predictions available in the literature (D'Arcy et al., 2006; Martinez et al., 2020). The results obtained here appear to be in general agreement with the simulation results obtained by those authors. However, different basket dimensions and operating conditions were used in the simulations. In other words, a quantitative comparison is challenging to establish without actual simulation data with correct dimensions of the system. Also, the simulations that those authors generated resulted in a perfectly symmetrical flow, as one could expect in this type of CFD simulations when geometric symmetry is imposed from the beginning. It should be remarked though that steady-state CFD simulations will always result in a perfectly symmetrical flow distribution, which will be difficult, if not impossible, to attain in real systems. It is also possible that more accurate time-dependent simulations may reveal in the future that flow instabilities are present even in perfectly symmetrical systems and are thus an intrinsic characteristic of this apparatus. Furthermore, since the velocities in Apparatus 1 appear to be significantly affected by small geometric features of the system, such as the basket clips, and by small asymmetries, it is likely that more accurate CFD simulations incorporating such characteristics of the apparatus in the CFD geometry will be required to obtain more accurate predictions. In any case, the experimental results obtained in this work are expected to be very relevant to validate the results of future computational predictions of the flow in Apparatus 1.

## 5 Conclusions

The following conclusions can be drawn from the work conducted in this study:

1. The velocity distribution in the USP Apparatus 1 appears to be extremely sensitive to even small deviations from perfect symmetry, requiring extensive and careful calibration of all components of the USP Apparatus 1 system to obtain reproducible velocity data;
2. The flow is dominated by the tangential component of the velocity on all horizontal planes. The flow in the tangential direction is nearly symmetrical around the vertical centerline in all cases. Nevertheless, despite all the precautions taken, small asymmetries in the flow could be observed on some horizontal planes, especially below the basket. This appears to be an unavoidable characteristic of the flow in USP Apparatus 1. The tangential velocities were always higher in the basket region and increased with agitation speed. Also, the tangential velocities increased with agitation speed in general and, in the hemispherical portion of the vessel, with increasing axial coordinate along the vessel height, reaching their highest value in the region adjacent to the basket. A low-velocity region near the central bottom region of the vessel could be measured at all agitation speeds, although this region decreased with increasing agitation speed;
3. The magnitudes of velocities on the vertical cross section through the vessel centerline were extremely low, typically below about 10 mm/s (compared to a maximum value of almost 50 mm/s in the horizontal planes) even at the highest agitation speed investigated here (100 rpm). Even on this plane the flow was not perfectly symmetrical. A small jet was seen emanating radially near the top of the basket. This flow is likely the result of the presence of the three-small clips holding the basket, which act as miniscule “impeller blades.” Despite the careful alignment of all components of the dissolution testing apparatus and the PIV system, small asymmetries in the flow on the vertical plane were always present. As one may expect, even minor deviations in the symmetry of the USP Apparatus 1 can result in small flow asymmetries on the vertical plane. These asymmetries were more evident for the radial and axial velocity components, because of their small magnitudes, than for the tangential velocities. One can additionally speculate that the small radial flow generated by an odd number of clips is likely to contribute to create small perturbations in the flow that propagate throughout the vessel contributing to breaking symmetry in a system already very gently stirred and dominated by the weak but, relatively speaking, much stronger tangential flow generated by the basket;
4. A detailed quantitative comparison of the components of the nondimensional velocity, scaled with the basket tip speed, showed that the actual velocities typically, but not always, scaled well with increasing agitation speed, implying that the agitation speed generally resulted in a nearly proportional increase in the velocities in most regions of the vessel, although some deviation from this general pattern could be noticed especially at specific locations and especially at low agitation speeds;
5. By measuring the axial velocities on a plane just below the basket it was possible to determine that *the velocities impinging the bottom mesh of the basket and penetrating directly into the basket scaled in directly proportionality to the agitation speed of the basket. This additionally implies that the flow through the basket bottom also increases proportionally to the basket agitation speed*, which, in turn, can be assumed to promote tablet suspension within the basket, increase the tablet-dissolution medium mass transfer rate, and, in general, promote faster tablet dissolution;
6. Having established that the hydrodynamics of the USP Apparatus 1 is extremely sensitive to even minute variations in the system geometry, such as those resulting from deviation from perfect symmetry even when the system is properly assembled, although without special precautions, it is reasonable to assume that similar small flow asymmetries would also be present during routine dissolution tests, although their impact on the variability of dissolution test results still remains to be determined;
7. To the best of our knowledge this is the first study quantifying experimentally the velocity distribution in USP Apparatus 1 in detail. The result of this work can guide industrial practitioners in their application in industry and lay the foundation for a more fundamental understanding, via possible eventual incorporation, of flow field data in the vessel into the prediction of the external mass transfer rate as related to the tablet dissolution process. Such knowledge would then help in the interpretation of dissolution testing data obtained under different geometries and operating conditions;
8. The results of this work should provide a major insight into the flow inside this apparatus. In addition, and despite the small flow asymmetries experimentally observed and quantified here, these results could be used to validate CFD solutions for the flow in the USP Apparatus 1, which could possibly be relatively inexpensively expanded to determine the flow inside the basket, and the effect of variations in geometric and operational parameters, as well as dosage form size and shape, on the flow field.
9. These results could additionally be used to support possible revisions of the USP-NF chapters dedicated to dissolution testing.

## USP identification of branded instruments or equipment disclaimer

Certain commercial equipment, instruments, vendors, or materials may be identified in this paper to specify adequately the experimental procedure. Such identification does not imply approval, endorsement, or certification by USP of a particular brand or product, nor does it imply that the equipment, instrument, vendor, or material is necessarily the best available for the purpose or that any other brand or product was judged to be unsatisfactory or inadequate.

## Declaration of Competing Interest

The authors declare that they have no known competing financial interests or personal relationships that could have appeared to influence the work reported in this paper.
